# The Effects of One-Stage Full-Mouth Disinfection and Qua-drant-Wise Scaling and Root Planing on Serum Levels of IL-17 and IL-1β and Clinical Parameters (A randomized Controlled Trial Study)

**Published:** 2013-05

**Authors:** Adileh Shirmohammadi, Zohreh Babaloo, Amir Eskandari, Reza Purabbas, Amirreza Babaloo

**Affiliations:** 1 Associated Professor, Dental and Periodontal Research center, Department of Periodontics, School of Dentistry, Tabriz University of Medical Sciences, Tabriz, Iran; 2 Assistant Professor, Department of Immunology, Tabriz University of Medical Sciences, Tabriz, Iran; 3 Assistant Professor, Department of Peiodontics, School of Dentistry, Tabriz University of Medical Sciences, Tabriz, Iran; 4 Professor, Department of Peiodontics, School of Dentistry, Tabriz University of Medical Sciences, Tabriz, Iran; 5 Assistant Professor, Department of Periodontics, School of Dentistry, Tabriz University of Medical Sciences, Tabriz, Iran

**Keywords:** Chronic Periodontitis; Disinfection; Dental Scaling/methods; Interleukin 17; Interleukin 1βeta

## Abstract

**Objective:** One-stage full-mouth disinfection technique (FMD) has been introduced to avoid cross-contamination between the treated and untreated regions between treatment sessions. Considering the role of inflammatory mediators in periodontitis, the aim of the present study was to compare the effects of FMD with the quadrant-wise scaling and root planing (Q-SRP) on serum levels of IL-17 and IL-1β in patients with moderate-to-severe chronic periodontitis.

**Materials and Methods:** Twenty patients with chronic periodontitis were selected randomly and based on inclusion criteria in each group. In order to evaluate the periodontal status, the clinical parameters of bleeding on probing (BOP), clinical attachment level (CAL), probing depth (PD) and modified gingival index (MGI) were measured and recorded before treatment and at 2- and 4-month intervals after treatment. Immunologic parameters of the study such as IL-17 and IL-1β serum levels were determined by special laboratory kits at the same intervals. Data were analyzed by SPSS 15 statistical software. Statistical significance was defined at p<0.05.

**Results:** The results showed a decrease in the means of IL-17 and IL-1β serum levels in both treatment modalities, with no statistically significant differences between the two study groups at the two time intervals (p>0.05). In the evaluation of periodontal parameters, all parameters exhibited clinical improvements in both groups, with no statistically significant differences between the two study groups (p>0.05).

**Conclusion:** Based on the results of the present study it was concluded that both FMD and Q-SRP techniques result in improvements in periodontal indexes and decreases in the serum levels of IL-17 and IL-1β inflammatory mediators.

## Introduction

Periodontitis is a chronic inflammatory disease in which an inflammatory process destroys thetooth-supporting structures and results in bone loss and periodontal pocket formation [1]. 

P gingivalis, T forsythia and Aggregatibacter are considered as the principal etiologic agents, but several other microorganisms are also involved in periodontal diseases [2-5]. The majority of these bacterial species are not only a component of subgingival plaque microbial flora, but they also colonize mucous membranes, the surface of the tongue and the tonsils and are usually found in saliva [6-10]. Since individual susceptibility to the disease cannot be altered at clinical levels (except for anti-inflammatory drugs), periodontal treatment focuses on decreasing or eliminating periodontal pathogens and the complete removal of the possibility of re-colonization (chiefly through pocket elimination by surgical procedures) and creation of an appropriate environment (with less anaerobic conditions) for pathogenic bacterial species. It should be noted that current therapeutic procedures are based on non-specific plaque hypothesis and eliminating useful bacterial species too [7-9]. 

However, only one week after treatment, the periodontal pocket is occupied by the same number of bacteria with low disease-inducing properties [11-12]. Proliferation of bacteria in subgingival areas [13] and junctional and pocket epithelia [14], or bacteria in the dentinal tubules, the dorsum of the tongue and on the tonsils might be possible sources of such subgingival colonization [15].

Based on the above-mentioned view, the one-stage full-mouth disinfection technique was introduced by the Belgian Lowen research group [16]. The aim of this technique is the rapid elimination or at least suppression of all periodontal pathogens from the oropharyngeal areas (periodontal pockets, saliva, oral mucosa and tonsils). In this technique, bacterial re-colonization through cross-contamination or intra-oral transportation is delayed until the appropriate healing of the periodontal pockets. The technique is rather new and there is a lot of controversy over its efficacy, rationale, patient and operator comfort, systemic effects and finally its cost-effectiveness. Therefore, a large number of clinical and paraclinical studies have been carried out on the subject, with contradictory results, making it difficult to make a sound judgment. Some studies have not reported any differences between the conventional quadrant-wise scaling and root planing procedure and the new technique [16-22]. Some others have reported that the new technique is superior to the conventional procedure [23-26]^.^

The majority of studies have clinically and microbiologically evaluated the new technique and only one study has evaluated the immunologic effect of the technique, reporting a significant decrease in pro-inflammatory mediators and an increase in anti-inflammatory mediators [27]. The research group which has introduced the technique believes modifications in the original technique are responsible for the discrepancies in the results of various studies [28].

Considering the discussion above, further studies are required to elucidate the various aspects of the new technique. An effective new method in the diagnosis and periodontal disease activity determination is measurement of serum biomarker levels [27,28]. The aim of the present study was to determine IL-17 and IL-113 serum levels to evaluate and compare the inflammatory processes in the two one-stage full-mouth disinfection technique and the quadrant-wise scaling and root planing procedure.

## MATERIALS AND METHODS

Forty patients (21 male, 19 female; age range, 25-62 years) referred to the department of Periodontics of Tabriz University of Medical Sciences were randomly allocated (by coin flip) to test (FMD) and control (Q-SRP) groups.

The subjects had at least 12 teeth (irrespective of third molars and teeth with orthodontic appliances, bridgeworks, crowns and implants) with 30% of the teeth with a clinical attachment loss of ≥3 mm and radiographic evidence of bone loss in each group, as specified by the inclusion criteria. The exclusion criteria included systemic conditions, conditions which delay wound healing, a history of radiotherapy or immune-suppressive treatment modalities, pregnancy or breast-feeling, use of systemic antibiotics during the two previous months, a history of continuous consumption of NSAIDs and a history of scaling and root planing during the previous year. 

Blood samples were provided before treatment and at 2- and 4-month intervals after treatment and sent to the laboratory for IL-17 and IL-1β serum level measurements with special kits. The quadrant-wise patients underwent scaling and root planing procedures at two-week intervals for each quadrant after the blood samples were taken. 

After blood samples were taken in the group undergoing FMD, the entire oral cavity was cleaned by an ultrasonic scaler in two sessions in less than 24 hours in order to decrease the pathogenic organism count. Subgingival irrigation of all the pockets was carried out with 0.2% chlorhexidine using an insulin syringe in order to remove all remaining bacteria. An antiseptic agent (0.2% chlorhexidine) was used to brush the dorsum of the tongue to decrease the bacterial load in the area; an antiseptic mouthwash (0.2% chlorhexidine) was used to reduce bacterial load in the saliva and on the tonsils. 

The variables under study included IL-17 and IL-1β serum levels and clinical parameters such as bleeding on probing (BOP) (a clinical inflammatory parameter indicating bleeding after probing), clinical attachment level (CAL) (a clinical parameter to determine the amount of support provided by periodontal tissues), probing depth (PD) (periodontal pocket depth as a result of periodontal attachment loss), and modified gingival index (MGI), which were measured before treatment and at 2- and 4-month intervals.

Plaque index was recorded using O’Leary method before treatment and at 2-, 4-, 8-, 12- and 16- week intervals after treatment with the aim of achieving an index of ≤10% (by instructing plaque control procedures) and maintaining it at that level. Gingival and plaque indexes were considered control variables in the present study and patients with inappropriate cooperation (high plaque index at periodic examinations) were excluded from the study.

***Statistical analysis***

All the measurements were performed by one calibrated periodontist who was blind to the treatment groups. Data were analyzed with descriptive statistical methods (means ± standard deviations) and parametric statistical tests, including comparison of means for dependent groups by paired samples t-test using SPSS 13 software. 

A chi square (X2) test was used to investigate whether distributions of categorical variables differ from one another. Paired sample t- test was used in order to compare the mean scores of case and control groups. Repeated measurement of ANOVA was applied to discover any probable differences within tested groups between the study timelines. A 0.05 statistically significance level was set to determine the meaningful differences.

## RESULT

Forty patients (21 male, 19 female) participated in the study. The average age of the FMD group was 43 ± 12.47 years and the average age of the Q-SRP group was 47.7 ± 9.4 years that was not statistically significantly different from the FMD group (p<0.05%). Statistical analysis showed that there was no statistically significant sexual difference among the participants and the sexual distribution between the groups (p<0.05%). 

The Kolmogorov-Smirnov test showed that all data in the study had a normal distribution (p>0.05). Table 1 shows clinical treatment outcomes and serum biomarkers within and between the treatment groups.

For all measurements, there was a statistically significant improvement between baseline and the later 2 and 4 months measurements in each group (p<0.05%). But the difference between 2 and 4 months was not statistically significant (p>0.05%). 

In the FMD group, the mean IL-1β serum level was 100.05 mm before treatment, which decreased to 60.25 and 54.38 pgr/ml at 2- and 4-month intervals, respectively after treatment.

**Table 1:** Clinical Treatment Outcomes and Serum Biomarkers Within and Between the Treatment Groups

	**FMD**	**Q-SRP**	**Between Groups**
PI Baseline 2 months 4 months	69.40 ±24.20	76.90 ±17.90	0.28
	12.20 ±19.31 ^†^	10.85 ±10.10 †	0.78
	5.45 ±3.25 ^†^	5.55 ±2.92 †	0.92
BOP Baseline 2 months 4 months	73.85 ±15.20	72.75 ±26.76	0.87
	17.90 ±9.31 ^†^	15.70 ±9.28 †	0.46
	12.75 ±13.68 ^†^	6.60 ±5.39 †‡	0.07
MGI Baseline 2 months 4 months	3.30 ±0.47	3.35 ±0.58	0.44
	1.80 ±0.76 ^†^	1.90 ±0.44 †	0.03*
	1.70 ±0.80 ^†^	1.80 ±0.52 †	0.04*
PD Baseline 2 months 4 months	4.18 ±0.58	4.17 ±0.66	0.99
	2.75 ±0.0.57 ^†^	2.74 ±0.62 †	0.93
	2.60 ±0.52 ^†‡^	2.56 ±0.55 †‡	0.84
CAL Baseline 2 months 4 months	4.20 ±0.57	4.19 ±0.66	0.99
	3.03 ±0.59 ^†^	3.02 ±0.62 †	0.93
	2.78 ±0.54 ^†‡^	2.74 ±0.55 †‡	0.84
IL-17 Baseline 2 months 4 months	359.39 ±252.27	353.32 ±246.75	0.84
	159.75 ±170.16 ^†^	154.20 ±171.53 †	0.45
	145.75 ±167.49 ^†‡^	139.78 ±163.97 †	0.24
IL-1*β* Baseline 2 months 4 months	100.05 ±63.73	96.61 ±27.86	0.53
	60.25 ±32.77 ^†^	70.15 ±20.07 †	0.10
	54.38 ±32.35 ^†^	61.60 ±21.38 †‡	0.28

^†^ Significant with Baseline

^‡^ Significant with 2 Months

Asterisk indicates statistical significance based on P<0.05.

There was statistically significant differences in IL-1β serum levels between the intervals of the study (p<0.01). 

In this group, the mean IL-17 serum level was 359.39 pgr/ml before treatment that decreased to 159.75 and 145.75 pgr/ml at 2- and 4-month intervals, respectively after treatment. 

There was statistically significant differences in IL-17 serum levels between the intervals of the study (p<0.05). In the Q-SRP group, the mean IL-1β serum level was 96.62 pgr/ml before treatment, which decreased to 70.15 and 61.61 pgr/ml at 2- and 4-month intervals, respectively after treatment.

There was significant differences in IL-1β serum levels between the intervals of the study (p<0.05). In the quadrant-wise scaling group, the mean IL-17 serum level was 353.32 pgr/ml before treatment that decreased to 154.20 and 139.78 pgr/ml at 2- and 4-month intervals, respectively after treatment. There was statistically significant differences in IL-17 serum levels between the intervals of the study (p<0.05).

***Comparison of data between the two techniques***

• IL-17 and IL-1β serum levels before treatment and 2- and 4-month intervals after treatment did not reveal any statistically significant differences between FMD and Q-SRP techniques (p>0.05).

• PI, PD, CAL and BOP parameters significantly decreased in both groups after the initial treatment (p<0.05); however, the differences in these parameters between 2- and 4-month intervals were not significant (p>0.05).

• PI, PD, CAL and BOP parameters exhibited no statistically significant differences before treatment and at 2- and 4-month intervals after treatment between the two techniques (p>0.05).

• MGI before treatment exhibited statistically significant differences between the two techniques (p<0.05).

## Discussion

In the present study, the effects of FMD and Q-SRP on IL-17 and IL-1β serum levels were evaluated and compared in patients with moderate-to-severe chronic periodontitis.

The results revealed a decrease in the means of serum levels of IL-17 and IL-1β subsequent to FMD and Q-SRP. None of the groups exhibited statistically significant differences in the means of IL-17 and IL-1β serum levels between the 2- and 4-month intervals after treatment. In addition, no significant differences were observed in the means of IL-17 and IL-1β serum levels between the two groups during the whole study period. In the evaluation of periodontal parameters of BOP, CAL, PD and MGI, clinical improvements were observed in both groups, with no significant differences between the two groups. The results showed improvements in clinical periodontal parameters along with a significant decrease in IL-17 and IL-1β serum levels in both disinfection techniques.

FMD technique was introduced by the Belgian Lowen research group [16]. The aim of this technique is rapid elimination or at least suppression of all periodontal pathogens from the oropharyngeal areas (periodontal pockets, saliva, oral mucosa and tonsils). In this technique, bacterial re-colonization through cross-contamination or intra-oral transportation is delayed until the appropriate healing of periodontal pockets. The technique is rather new and there is a lot of controversy over its efficacy, rationale, patient and operator comfort, systemic effects and finally its cost-effectiveness. Therefore, a large number of clinical and paraclinical studies have been carried out on the subject, with contradictory results, making it difficult to make a sound judgment. An effective new method in the diagnosis and periodontal disease activity determination is measurement of serum biomarker levels [27,28]. In the present study, the inflammatory process of periodontal diseases was evaluated for the first time by determining IL-17 and IL-1β serum levels before and after two disinfection techniques of FMD and Q-SRP. To the best of our knowledge, this is the first study to evaluate the above-mentioned immunologic markers before and after the application of the two disinfection techniques. 

IL-17 is a pro-inflammatory cytokine, which is secreted by T-calls, predominantly by Th1/Th0 phenotypes. Vernal et al. showed high levels of IL-17 in the GCF in patients with periodontitis and emphasized the role of this cytokine in the pathogenesis of chronic periodontitis [29]. It has been reported that outer membrane proteins of *P gingivalis* induce a significant increase on IL-17 secretion in periodontitis patients and IL-17 is more commonly seen in periodontitis rather than gingivitis patients [30]. IL-1 has a direct relationship with probing depth and attachment loss [31,32]. Goutoudi et al. evaluated levels of the pro-inflammatory IL-1β in the GCF of patients with chronic periodontitis before periodontal treatment. The results revealed high levels of this cytokine in patients with periodontitis and the relationship between its serum levels and disease severity [33]. Some studies reported no significant differences between the two disinfection techniques [16-22] and some others have emphasized the superiority of the new technique over the conventional treatment modality [23-25].The majority of studies have evaluated the clinical and microbiological aspects of the new technique and only one study has evaluated the immunologic effects of this technique, reporting a significant decrease in pro-inflammatory mediators and an increase in anti-inflammatory mediators [27]. Apatzidou et al. evaluated the effect of FMD techniques on antibody titers and reported that both treatment modalities resulted in a decrease in antibody titers during a six-month period, with no significant differences between the two techniques [27], consistent with the results of the present study. In other words, both treatment modalities induced a decrease in serum levels of IL-17 and IL-1β during a 4-month period in the present study. The research group that has introduced the technique believes modifications in the original technique are responsible for the discrepancies in the results of various studies [28].

Considering what was discussed above, further studies are required to shed light on various aspects of the new technique.

## CONCLUSION

Based on the results of the present study, both one-stage full-mouth and quadrant-wise disinfection techniques are equally effective in improving periodontal parameters. In both techniques, a decrease in the serum levels of IL-17 and IL-1β pro-inflammatory cytokines along with improvements in clinical periodontal parameters was observed during the study period. 

Considering the advantages of one-stage full-mouth disinfection technique, patient and operator comfort, systemic effects and its cost-effectiveness, the use of this technique in periodontitis patients is strongly recommended.
